# High-Performing and Low-Performing Hospitals Across Medicare Value-Based Payment Programs

**DOI:** 10.1001/jamahealthforum.2022.1864

**Published:** 2022-07-22

**Authors:** Dhruv Khullar, Wei Tian, Rishi K. Wadhera

**Affiliations:** 1 Division of Health Policy and Economics, Department of Population Health Sciences, Weill Cornell Medical College, New York, New York; 2 Section of Health Policy and Equity at the Richard A. and Susan F. Smith Center for Outcomes Research in Cardiology, Beth Israel Deaconess Medical Center, Boston, Massachusetts

## Abstract

This cross-sectional study examines the number and characteristics of hospitals that performed well or poorly across 3 Medicare value-based programs in fiscal year 2020.

## Introduction

Over the past decade, the Centers for Medicare & Medicaid Services (CMS) has introduced 3 major hospital value-based payment programs that aim to improve quality: the Hospital Value-Based Purchasing Program (HVBP), the Hospital Readmissions Reduction Program (HRRP), and the Hospital-Acquired Condition Reduction Program (HACRP). Hospitals penalized by all 3 programs may experience a reduction in Medicare revenue of up to 6%—a major loss, given that many US hospitals operate on narrow margins.^1^ Given the substantial financial implications, understanding how many US hospitals currently perform poorly across all 3 programs and the characteristics of the communities served by these sites is important. Prior work has shown that each program disproportionately penalizes hospitals caring for patients from low-income and racial and ethnic minority groups, raising concern that aggregate penalties may impede their ability to provide and improve care.^2,3,4^ At the same time, understanding the types of hospitals that perform well across all programs could inform strategies to improve care delivery.

## Methods

In this cross-sectional study, we examined the number and characteristics of hospitals that performed well or poorly across all 3 value-based programs in fiscal year (FY) 2020 (which reflects performance between July 1, 2015, and June 30, 2018, for HVBP and HRRP, and July 1, 2016, and June 30, 2018, for HACRP). Using Medicare’s Hospital Compare files, we identified the FY 2020 scores of hospitals participating in the HVBP, HRRP, and HACRP. Hospital characteristics were obtained from the American Hospital Association Annual Survey and CMS Impact File, and county characteristics from the American Community Survey File. In each program, we categorized hospitals into performance quartiles and identified hospitals that ranked in the top (high-performing) and bottom (low-performing) quartiles across all 3 programs, and classified all other hospitals as average-performing. Cochran-Armitage test (binary variables) and linear regression (continuous variables) were used to assess for monotonic patterns across hospital groups, and Cochran-Mantel-Haenszel tests for mean differences (categorical variables). Two-sided *P* < .05 was considered statistically significant. All analyses were performed using R, version 3.5.2 (R Foundation for Statistical Computing). The Beth Israel Deaconess Medical Center institutional review board deemed this study exempt because it used only publicly available data without patient identifiers. This study adhered to the Strengthening the Reporting of Observational Studies in Epidemiology (STROBE) reporting guideline for cross-sectional studies.

## Results

In FY 2020, 2725 hospitals participated in all 3 payment programs. Of these hospitals, only 87 (3.2%) were identified as high-performing sites, while 74 (2.7%) had low performance across all 3 programs; the proportion receiving penalties in each hospital group is shown in the Table. Low-performing hospitals were larger than average-performing and high-performing hospitals (mean hospital beds: 313.7 vs 241.1 vs 139.7; *P* < .001), more likely to be for-profit (41.9% vs 20.4% vs 13.8%; *P* < .001), and similarly likely to be safety-net hospitals (18.9% vs 25.9% vs 19.5%; *P* = .95) (Table). Low-performing hospitals were more likely than average-performing and high-performing hospitals to be located in counties in which a higher proportion of the population was comprised of Black (18.6% vs 11.9% vs 6.1%; *P* < .001) or non-US-born (11.9% vs 11.0% vs 8.1%; *P* = .01) adults and were concentrated in states in the South Atlantic (27.0%) and East South Central (17.6%) regions (Figure). Across hospital groups, there were no statistically significant differences in teaching status or in the county-level proportion of individuals living in poverty or with a bachelor’s degree.

**Table.** Characteristics of Low-Performing, Average-Performing, and High-Performing US Hospitals Across 3 Value-Based Programs^a^^,^^b^

Characteristic	Hospitals, No. (%)			*P* value
	Low-performing hospitals (n = 74)	Average-performing hospitals (n = 2533)	High-performing hospitals (n = 87)	
**Hospital characteristics**				
No. of hospital beds, mean (SD)	313.7 (186.8)	241.1 (225.4)	139.7 (137.5)	<.001
Ownership				
For profit	31 (41.9)	517 (20.4)	12 (13.8)	<.001
Private nonprofit	36 (48.7)	1679 (66.3)	69 (79.3)	
Public	7 (9.5)	339 (13.4)	6 (6.9)	
Rural location	3 (4.1)	134 (5.3)	10 (11.5)	.03
Teaching hospital	43 (58.1)	1296 (51.2)	40 (46.0)	.13
Safety-net status^c^	14 (18.9)	656 (25.9)	17 (19.5)	.95
US region				
Northeast	5 (6.8)	114 (4.5)	5 (5.8)	<.001
Middle Atlantic	11 (14.9)	299 (11.8)	6 (6.9)	
South Atlantic	20 (27.0)	443 (17.5)	8 (9.2)	
East North Central	8 (10.8)	410 (16.2)	24 (27.6)	
West North Central	1 (1.4)	200 (7.9)	10 (11.5)	
East South Central	13 (17.6)	223 (8.8)	3 (3.5)	
West South Central	7 (9.5)	334 (13.2)	6 (6.9)	
Mountain	5 (6.8)	172 (6.8)	7 (8.1)	
Pacific	4 (5.4)	342 (13.5)	18 (20.7)	
**County characteristics, mean (SD), %**				
Living in poverty	15.9 (5.2)	14.9 (5.4)	14.4 (6.1)	.08
Black or African American^d^	18.6 (15.2)	11.9 (12.9)	6.1 (9.1)	<.001
Non-US born	11.9 (11.5)	11.0 (10.1)	8.1 (8.5)	.01
Population aged ≥25 y with a bachelor’s degree or higher	29.7 (10.1)	29.6 (11.1)	28.3 (11.9)	.39
**% Penalized by value-based programs^e^**				
HACRP	100	24.6	0	<.001
HRRP	100	89.0	44.8	<.001
HVBP	100	44.0	0	<.001
All 3 programs	100	12.6	0	<.001

Abbreviations: HACRP, Hospital-Acquired Condition Reduction Program; HRRP, Hospital Readmissions Reduction Program; HVBP, Hospital Value-Based Purchasing Program.

^a^ Hospitals in the top quartile of performance of the HVBP, HRRP, and HACRP were classified as high-performing, and those in the lowest quartile of performance across all 3 programs were classified as low-performing. A total of 2725 were eligible for payment adjustments in all 3 programs in fiscal year 2020.

^b^ Fiscal year 2020 reflects performance based on data between July 1, 2015, and June 30, 2018, for the HVBP and HRRP and July 1, 2016, and June 30, 2018, for the HACRP.

^c^ Safety-net hospitals were defined as those in the highest quartile of the Disproportionate Share Hospital Index.^2^

^d^ Race was determined for county characteristics.

^e^ The proportion of low-performing, average-performing, and high-performing hospitals receiving financial penalties under each value-based program, as well as the proportion in each group receiving penalties from all 3 programs in fiscal year 2020.

**Figure.**
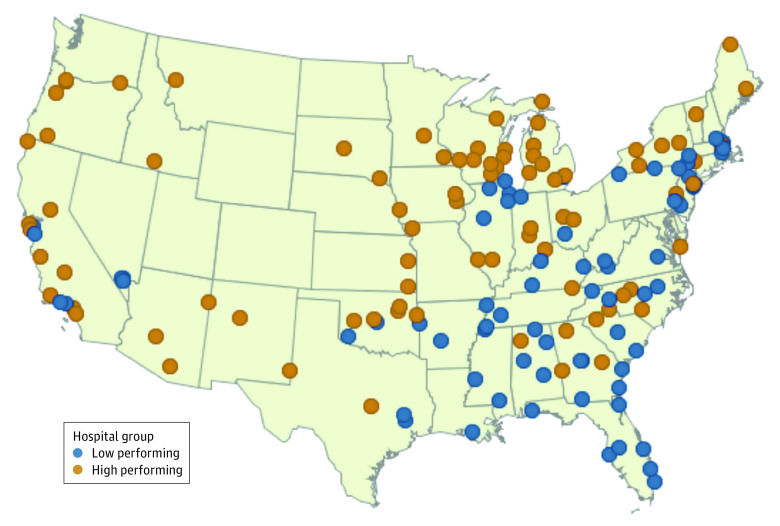
Low-Performing and High-Performing US Hospitals Across All 3 Value-Based Programs The map shows the geographic distribution of low-performing (blue) and high-performing (orange) hospitals across 3 hospital value-based programs (Hospital Value-Based Purchasing Program, Hospital Readmissions Reduction Program, Hospital-Acquired Condition Reduction Program) in the US. Hospitals that performed poorly across all 3 programs were more likely to be located in the Southeast. Four hospitals located in Alaska and Hawaii are not shown on the map.

## Discussion

Less than 5% of US hospitals demonstrate high performance across all 3 of Medicare’s hospital value-based payment programs. This could reflect efforts by CMS to capture separate domains of quality and would imply that very few US hospitals provide high-quality care globally. Alternatively, it may result from idiosyncrasies of program design and measures that hamper reliable assessment of hospital quality and that may warrant attention and reform.^5,6^

Hospitals that performed poorly across programs were more likely to be located in areas with a higher proportion of socially vulnerable populations, including immigrants and racial and ethnic minority groups. Nearly half of low-performing hospitals were located in the Southeastern US, where many communities experience disproportionately poor health outcomes. Financial penalties levied by hospital value-based programs, particularly when compounded, may exacerbate disparities in care for these populations.

A limitation of this study is that we only focused on FY 2020, which reflects hospital performance over a 3-year period (2015 to 2018).

Very few hospitals demonstrate high performance across all 3 value-based programs (HVBP, HRRP, HACRP), and hospitals penalized by all 3 programs (low performers) were more likely to be located in communities with a high proportion of Black individuals and in the Southeastern US.
